# The Psychosocial Impact of Neurobehavioral Disability

**DOI:** 10.3389/fneur.2020.00119

**Published:** 2020-02-20

**Authors:** Claire Williams, Rodger Llewellyn Wood, Nick Alderman, Andrew Worthington

**Affiliations:** ^1^Department of Psychology, College of Human and Health Sciences, Swansea University, Swansea, United Kingdom; ^2^Swansea University Medical School, Swansea University, Swansea, United Kingdom; ^3^Elysium Neurological Services, Elysium Healthcare, Badby Park, Daventry, United Kingdom; ^4^Headwise Limited, Birmingham, United Kingdom

**Keywords:** neurobehavioral disability, social cognition, empathy, apathy, aggression, brain injury, psychosocial outcome

## Abstract

Neurobehavioral disability (NBD) comprises elements of executive and attentional dysfunction, poor insight, problems of awareness and social judgement, labile mood, altered emotional expression, and poor impulse control, any or all of which can have a serious impact upon a person's decision-making and capacity for social independence. The aim of this narrative review is to explore some of the more intrusive forms of NBD that act as obstacles to psychosocial outcome to act as a frame of reference for developing effective rehabilitation interventions. Special consideration is given to the psychosocial impact of three core forms of NBD: a failure of social cognition, aggressive behavior, and problems of drive/motivation. Consideration is also given to the developmental implications of sustaining a brain injury in childhood or adolescence, including its impact on maturational and social development and subsequent effects on long-term psychosocial behavior.

## Introduction

Neurobehavioral disability (NBD) is often considered a legacy of traumatic brain injury (TBI) but can follow any kind of brain injury, usually when the frontal system of the brain is compromised in some way. NBD is the product of an interaction between damaged neural systems, neurocognitive impairment, and environmental factors, further influenced by pre-morbid personality traits, post-injury learning, and a variety of environmental influences (1–3). It can take many forms, some of which involve a lack of social cognition (often involving problems of emotion-recognition and expression), or a lack of inhibitory control (such as labile mood, impulsivity, low tolerance, irritability, and poor temper control), while other forms present as diminished patterns of behavior (characterized by a lack of arousal-drive-motivation). When brain injury occurs during childhood or adolescence many forms of NBD can be more subtle, yet have a pervasive influence on maturational and social development. It is also the case that many aspects of NBD are not apparent in the early recovery stages after brain injury, only becoming evident when the injured person leaves a hospital or rehabilitation setting (both of which are highly structured environments) and have to begin to organize their lives, make decisions, re-establish relationships, and settle back into a constructive routine conducive to community independence.

NBD can act as a major obstacle to psychosocial recovery by undermining a person's capacity for independent social behavior and employment opportunities. Alterations to behavior and personality are long-lasting and enduring. They act as a significant barrier to making and sustaining relationships [e.g., (4)] and can impose a serious level of stress upon families who often struggle to adapt to life with a relative who exhibits altered patterns of behavior (5–9). As time passes, relatives start to experience an increasing sense of “burden” (10), often because they are unaware of the neurobehavioral implications of brain injury and are unprepared for the emotional demands of the caregiving role (11, 12). Relatives experience a lack of control in their life planning (since they cannot schedule their activities) and uncertainty regarding their future because of ambiguity regarding their caregiving role (13–17).

Therefore, in order to provide an effective post-acute rehabilitation structure to maximize psychosocial recovery, knowledge of the nature and potential impact of NBD is vital in order to set meaningful rehabilitation goals, understand the probable time needed to achieve such goals, and indeed, whether the goals are realistic, considering a person's type or degree of disability. This review is not a systematic review about the topic, but a narrative overview that aims to raise awareness of some of the more intrusive forms of NBD and their potential psychosocial impact in order to provide a perspective for an effective rehabilitation framework.

## A Failure of Social Cognition

Social cognition refers to the ability to attend to, recognize, interpret, and respond appropriately and flexibly to social cues that guide social behavior. Hence it is a broad construct in which different components can be distinguished. McDonald (18) made a distinction between “*hot processes,”* including emotion perception and the ability to empathize, and “*cold processes,”* which reflect the ability to infer the beliefs, feelings, and intentions of others (e.g., Theory of Mind—ToM) in order to see their point of view (cognitive empathy) and what they mean when communicating (pragmatic inference). Thus, social cognition consists of different and dissociable, but interrelated processes (19).

Social cognition appears to be underpinned by a frontal sub-cortical network, including orbital and ventromedial regions (20, 21), the cingulate cortex and striatum, insula, and amygdala (22, 23); structures particularly vulnerable to traumatic brain injury (TBI) either due to focal, multifocal or diffuse axonal injury (24–26). Consequently, it is perhaps unsurprising that impairments in social cognition have been frequently observed after TBI, including deficits of emotional perception and recognition [e.g., (27, 28)] and ToM (29, 30), which often culminate in a lack of empathy (31).

### Diminished Empathy

Empathy involves three primary components: *cognitive empathy*—the ability to appreciate and understand how and why a person exhibits an emotional state; *emotional empathy*—the capacity to vicariously experience and share the perceived emotional experiences of others (31); and *compassionate empathy*; an appraisal mechanism that keeps track of feelings experienced by oneself and others, allowing one to decide whether it's appropriate to respond compassionately (32). Therefore, empathy requires the ability to share (emotional empathy) or understand (cognitive empathy) another's emotional state and then feel concerned about that person's welfare (compassionate empathy).

In a sample of 89 individuals with TBI, Wood and Williams (33) found that 60.7 per cent reported low levels of emotional empathy, compared with only 31 per cent of a demographically matched healthy control group. Interestingly, they found no significant relationships between emotional empathy and cognitive abilities (i.e., cognitive flexibility, executive function, verbal ability), suggesting that emotional empathy may operate in a manner that is relatively independent of cognitive ability *per se*. They also found no obvious relationship between emotional empathy and measures of affective distress. Finally, evidence suggests that emotional empathy is unrelated to the severity of TBI (31, 33), implying that even relatively minor head injury (presumably in vulnerable individuals) has potential to disrupt the capacity to empathize.

### The Role of Alexithymia

One such vulnerability factor is alexithymia, a multifaceted construct comprising: (a) difficulty identifying and describing emotions; (b) a concrete communication style; (c) an externally oriented style of thinking, and (d) limited imaginal capacity (34). Clinically, individuals exhibiting alexithymia demonstrate little knowledge about their own feelings and, in most instances, are unable to link them with memories, fantasies, higher level affects, or specific situations (35). It is a normally distributed personality trait present in 7-12% of the population (36, 37) and, whilst not intrinsically pathological, it has been conceptualized as one of several personality risk factors for a variety of medical and psychiatric disorders involving problems of affect regulation [For a review, see (38)].

Recent studies have revealed a much higher incidence (57.4–72.3 per cent) of alexithymia after TBI (31, 39–41), with the terms “organic” and “acquired” alexithymia subsequently adopted to descriptively distinguish constitutional deficits associated with a developmental history of affective and personality disorders, from an acquired disorder following TBI. Further, Williams and Wood (31) found an inverse relationship between alexithymia and emotional empathy in 64 cases with TBI and 64 demographically matched healthy controls, suggesting that the presence of alexithymia may render an individual unable to vicariously experience the emotions of others (emotional empathy).

### The Impact on Social Behavior and Relationships

Behaviorally, a lack of empathy after TBI is often observed via a lack of social tact and social discretion, selfish and socially immature behavior (17, 42), an egocentric, self-centered attitude that is insensitive to, or neglectful of, the needs of others (3, 43), and a lack of emotional affection and relational connection with loved ones (44, 45). Unsurprisingly, a lack of empathy after TBI can therefore contribute to the fragility of close personal relationships when a partner, who was previously loving and affectionate, remains physically present but psychologically absent, emotionally withdrawn, and aloof after their injury. Indeed, it is not uncommon for spouses of individuals with TBI to describe their partners as a “*complete stranger”* since their injury, with their relationship no longer feeling like a marriage (8, 44, 46).

Close personal relationships following TBI therefore appear to be particularly vulnerable to strain and breakdown, with the rate of divorce typically higher than general population estimates of 22–42 per cent (47). For example, Wood and Yurdakul (48) found that 49 per cent of their UK sample of 131 couples had divorced or separated during the 5–8 year period following injury, with the rate of divorce increasing over the passage of time. This was assumed to indicate partners progressively losing hope that their loved one will recover and a realization that the permanence of the condition can no longer be denied. However, more recent examination of marital stability after TBI presents a stark contrast to earlier reports (49, 50). In a sample of 120 patients with mild to severe injuries, Kruetzer et al. reported a similar rate of divorce (17 per cent) as found by Wood and Yurdakul, but a much lower separation rate (8 per cent). This can be explained in part by a lack of consistency in assessment procedures, partly by the large socio-cultural differences between study samples, and partly on the basis of time since injury. For instance, Wood and Yurdakul followed-up cases 5–8 years post injury, concluding that 5 years post injury was a watershed for couples deciding to separate. By contrast, the Kreutzer study followed-up cases 2.5–7 years post-injury, potentially including a number of cases who had not yet reached the watershed point.

Despite uncertainty concerning divorce and separation rates following TBI, relationship stability and quality is generally reported to be low (4, 40). Peters et al. (51) found that partners of individuals with severe TBI reported significantly lower levels of marital satisfaction, cohesion, adjustment, and affectional expression compared to partners whose spouse had sustained a spinal cord injury. Similarly, Gosling and Oddy (52) reported significantly poorer marital satisfaction, plus a lack of expressed affection and emotional responsiveness in couples 1–7 year's post-TBI. They also noted that the non-injured partner was often more dissatisfied with their relationship than their injured spouse, a finding also supported by Williams and Wood (40). This highlights how many individuals with TBI lack awareness and insight into the impact of their injury on their significant others (53, 54).

The high rates of relationship breakdown and dissatisfaction following TBI described above have been linked to a wide range of factors, including changes in behavior, personality and neuropsychological function (4, 55), sociodemographic factors (56, 57), specific relationship factors [i.e., length of relationship; (48, 58)], and injury-related variables, such as severity of injury or time since injury (49). However, for many partners and spouses of individuals with TBI, the most challenging and destructive relationship behaviors following injury include a loss of emotional responsivity, a lack of mutual emotional support and companionship, and a loss or reduction in overt acts of affection. For example, relationships can suffer when individuals with TBI lack understanding of other people's social behavior and intentions (30), appearing insensitive or indifferent to the emotional needs of loved ones as a result.

Similarly, close personal relationships can be further compromised when partners or spouses report a sense of rejection, emotional isolation and detachment. This can occur if the relative to whom they provide care and support lacks empathy and seems emotionally cold and distant toward them, in contrast to their pre-accident behavior (33). This may create a feeling on the part of the relative that the support they give is not valued, a perception thought to be a significant contributor to both an objective and subjective sense of burnout. Sundin et al. (59) have also found that perceptions of poor appreciation from others involved in one's work or care role predicted emotional exhaustion, feelings of depersonalization, and a sense of poor personal accomplishment. In addition, Wells et al. (60) found that a lack of empathy on the part of the survivor uniquely contributed to a reduction in perceived life satisfaction in their sample of caregivers, of whom the majority were spouses.

How a spouse reacts to their partner's lack of empathy, and other stressors attached to their caregiver role [see (61)], will invariably depend on how they themselves experience and/or reflect on their own emotions. For instance, the presence of alexithymia has been positively correlated with higher levels of burnout and emotional exhaustion, as well as negatively correlated with feelings of personal achievement in occupational and professional samples [e.g., (62, 63)]. Similarly, in a sample of relatives of individuals who had sustained a TBI, Katsifaraki and Wood (64) found higher levels of emotional exhaustion and depression, as well as reduced levels of self-accomplishment in a sub-group of relatives reporting alexithymia, than a sub-group without alexithymia. More broadly, Mattila et al. (65) also found that difficulty identifying feelings (a core component of alexithymia), was associated with occupational burnout after controlling for depression and various sociodemographic factors. They also noted that alexithymic individuals were prone to burnout because of the adoption of dysfunctional coping mechanisms in order to deal with stressors, an observation also made by Demerouti et al. (66) and Parker et al. (67), as well as Wood and Doughty (68) in the context of TBI specifically.

### Risks of Social Isolation

In addition to close personal relationships, poor emotional perception, leading to a lack of emotional expression and an empathic response, may also help to explain why a high proportion of individuals with TBI experience deficiencies in social-interpersonal behavior that lead to a decline in social and leisure activities, diminishing social networks, poor community integration, and high levels of social isolation and loneliness (69–73). For instance, May et al. (74) found that poor emotion recognition abilities post-injury were associated with poor social functioning and fewer independent social activities outside of the home (i.e., community integration). An association has also been made between empathy and interpersonal behaviors that can directly, or indirectly, undermine social competence. For instance, Saxton et al. (75) found that perspective taking deficits (a critical component of empathy) were significantly related to both self-reported interpersonal (i.e., difficultly getting along with other people; getting into arguments easily) and communication problems, such as failing to listen carefully and respond normally when talking to others. From this perspective, it is likely that difficulty understanding another person's point of view restricts an individual's ability to effectively interact with others, such as exhibiting poor listening skills, misinterpreting the behavior of others and responding inappropriately (i.e., temper outbursts, emotional lability), failure to adjust behavior in accordance with social rules and demands, or communicating information that is irrelevant, insensitive, or redundant to the social situation. Consequently, such interpersonal and communication patterns are likely to alienate others, exerting an adverse impact on the size or quality of a person's wider social network.

In such circumstances individuals have fewer opportunities to observe and practice appropriate social-interpersonal communication, helping to explain the relative temporal stability and persistence [i.e., (76)] of such social-interpersonal difficulties over a long period of time post-injury. If the availability of meaningful social feedback is reduced it may add to lost opportunities to learn from experience, potentially leading to further social ineffectiveness and isolation, difficulty establishing new social contacts and friends, heightened feelings of failure, frustration and low self-esteem, and increased dependence on family members for social interaction and access to community and recreational activities. Crucially, this increased reliance on family members places additional strain on what are already fragile relationships, exacerbating the risk of relationship failure (4, 40) as well as caregiver stress, burn-out and psychological distress (61, 77). Therefore, in situations where close personal relationships dissolve after brain injury, the individual may not only have to come to terms with the loss of a partner, but also their primary, and potentially last, remaining source of social and emotional support.

Social isolation and lost social supports have additionally been linked to the adoption of maladaptive coping strategies that may further undermine recovery after TBI. For example, those who are socially isolated after TBI are more vulnerable to alcohol and drug dependency because of both their perceived direct mood-altering effects and the desire to compensate for a lack of socially meaningful and fulfilling contact with others [see (78, 79)]. This is particularly problematic as the behavioral and emotional effects of such substances and TBI are considered synergistic, leading to further negative impacts on an individual's social-interpersonal and psychosocial recovery.

## Aggressive Behavior

Aggression is arguably the most overt and debilitating feature of NBD (80) because of the serious impact it has on the survivor, their family and community. When aggressive behavior occurs in the context of rehabilitation, it can also prevent survivors achieving their full recovery potential (81), with some excluded from rehabilitation altogether. When this happens, individuals with brain injury gravitate to placements ill-equipped to meet their needs, including forensic and secure mental health services (82).

However, there is considerable variability regarding its prevalence. For example, in a review of the literature, Tateno et al. (83) found that rates of aggressive behavior amongst samples of TBI survivors varied from 11– 96 per cent. This extreme variability is partly attributable to the non-homogeneous nature of ABI, the lack of a standardized definition of what constitutes aggression, and methodological issues regarding how and when, or in what context, it is measured.

### Measuring Aggressive Behavior

Our understanding of aggression and its impact on psychosocial outcomes after brain injury can be improved by using standardized measures which incorporate clear, objective operational definitions of behaviors, such as those included in observational recording measures, like the “Overt Aggression Scale—Modified for Neurorehabilitation” [OAS-MNR: (84)] and the “Overt Behavior Scale” [OBS: (85)], both of which share the same operational definitions of different types and severity of aggression. When using such measures, prevalence rates between 40 and 90 per cent have typically been reported, with verbal aggression accounting for the greatest proportion. For example, using the OBS, Kelly et al. (86) investigated challenging behavior profiles of people who had suffered brain injury in the community, reporting that 85 per cent had engaged in verbal aggression, 41.1 per cent physical assaults on other people, and 35.3 per cent physical aggression toward objects. Some of the variability in prevalence reflects how long after injury data was captured, as the tendency is for aggression to increase over time. In addition, the impact of context on behavior is also evident from studies reporting on the prevalence and impact of aggression on psychosocial function in residential and hospital settings, which might typically be expected to manage those TBI survivors with the most challenging behavior. For example, Alderman et al. (87) described 5,548 aggressive events, including 729 physical assaults on other people, exhibited by 108 ABI survivors engaged in neurobehavioral rehabilitation over a 14 day period.

### Psychosocial Impact

However, aggressive behavior, like many other features of NBD can have complex origins so predicting its psychosocial impact is not straightforward. Accounts typically discriminate between aggressive behavior which has a predominantly neurological basis from behavior that is primarily attributable to neurocognitive impairment (88). Consequently, it is important to understand how these two forms impact on psychosocial function in order to devise effective rehabilitation interventions.

Briefly, and regarding neurological causes, lesions to the orbitofrontal cortex and its connections are especially implicated in aggression. Damage to the orbito-temporal-limbic feedback loop disrupts the inhibitory function of the cortex over the amygdala, depriving the cognitive functions of any ability to suppress instinctive emotional reactions (89). Aggressive behavior with this etiology is provoked by clear antecedents. A further category of neurologically mediated aggression is the episodic dyscontrol syndrome (EDS), one of the post-traumatic temporo-limbic disorders. EDS aggression tends to be brief, and ‘out of character’, often without obvious triggers. If there is some form of trigger it is usually minor and the magnitude of the behavioral response is grossly out of proportion. Whilst those with EDS often express regret over their behavior (usually in contrast to those who exhibit impulsive aggression), the unexpected nature of the aggressive outburst can have an extremely adverse emotional impact upon families (3).

Regarding neurocognitive impairment, executive function disorders are especially implicated in aggression. Reduced ability to initiate or use preserved abilities, monitor performance and utilize feedback effectively to regulate behavior, results in lack of “error awareness,” usually observed as disinhibition, impulsiveness and poor response to cues. This has a grossly negative impact on psychosocial function, incapacitating performance in social situations, reflected by low frustration tolerance and little ability to inhibit aggressive responses (88). This type of aggression is often underpinned by a form of procedural learning, especially when aggression serves an avoidance/escape function (90).

Irritability is similarly more evident amongst people with brain injury, especially TBI (91) and is strongly associated with overt aggression. Reports of incidence vary [for example, 29–69% in TBI, see (92)] and, as is also the case with aggression, some of this variance is attributable to lack of a standardized definition. However, most sources define irritability as involving an internal experience (becoming easily annoyed, upset) as well as overt expressions reflecting that experience (88). Irritability and aggression have been conceived as comprising opposite ends of a continuum; upsurges in irritability increase the likelihood of aggressive acts, with a variety of mediators underpinning movement along the continuum. For example, there is some evidence that severity of injury may act as one such mediator. Yang et al. (91) found a strong association between irritability and information processing ability amongst mild TBI survivors, which may be an important antecedent to aggressive acts described earlier regarding neurocognitive impairment and lack of error awareness. Conversely, they found no such association in survivors of moderate and severe TBI, concluding that irritability was a direct consequence of the brain lesions involved. As described above, this would be consistent with the association of aggressive behavior with lesions to the orbitofrontal cortex and its connections, and the subsequent deprivation of the cognitive functions in supressing emotional reactions.

### Impact on Community Living

There is a negative association between increased levels of challenging behavior, including aggression, with decreased levels of functional ability, increased care needs and decreased participation in life roles amongst TBI survivors in the community (93). For example, aggression features frequently in qualitative studies capturing the perspective of family members, many of whom attribute much of the decline in psychosocial function to this behavior. Braine (94) found that relatives commonly identified increased aggression and memory disturbance as being responsible for a number of negative experiences, including emotional turmoil, social isolation and concern for the future. Fear of behavioral outbursts was of particular concern. Similarly, Tam et al. (95) interviewed caregivers of severe TBI survivors in the community, finding that verbal outbursts, amongst a broad range of other challenging behaviors, were frequently cited as a significant concern. Distress and caregiver burden was especially highlighted with the additional consequence of reduced community integration for TBI survivors.

Gould et al. (96) interviewed TBI survivors either living at home or in residential accommodation, their close others, and clinicians regarding the impact of challenging behavior, including aggression. Some differences were found. Verbal and physical aggression were characteristic of survivors in the community, who were also found to have awareness of these behaviors and their psychosocial impact. In residential settings there was a broader range of challenging behavior, including violence that was more severe than that exhibited in the family home. This group also tended to lack awareness regarding their behavior and its consequences. Verbal aggression took the form of shouting, swearing and threats of physical violence, often alongside acts of physical aggression. Frustration and loss of control were reported as underpinning much of this. By contrast, aggression displayed within the home setting was more often associated with socializing or in formal interactions with authority figures, including the police. Aggressive reactions reflected impairments in social cognition, whereas in residential settings, the main cause of aggression was being prompted to perform personal care tasks. Aggression and concern about the unpredictability of violence was also noted to be very distressing to relatives, along with fear of the consequences of this behavior (police involvement and incarceration). Factors that triggered aggression included: a) a lack of routine and consistency (especially in residential settings); b) mental health problems (especially depression and anxiety); c) increased awareness, and d) a lack of meaningful activity. In addition, aggression in residential settings potentially served a number of additional functions, including attracting attention, avoiding activities and regaining control.

### Risk of Offender Behavior

Williams et al. (97) found that the tendency to react aggressively is associated with an increased risk of offender behavior and contact with forensic services, evidenced by the finding that individuals with TBI are overrepresented in UK prison populations (see later section on developmental implications). Associations between aggression and offending after TBI have also been reported in large scale Swedish population studies [e.g., (98)]. For example, Fazel et al. (99) demonstrated that violent crime was over-represented amongst people with TBI compared to the general population (8.8 vs. 2–3 per cent).

## Disorders of Arousal, Drive and Motivation

Motivation is essential to adaptive functioning and quality of life. Clinicians know that without motivation, individuals with TBI will fail to keep appointments, neglect their medications, become distant to friends and family, or fail to return to work. A lack of motivation imposes constraints on physical rehabilitation and the development of coping skills. It can also be an important source of burden for families who care for individuals after TBI (12).

The terms *arousal, drive* and *motivation* represent a continuum of psychophysiological function which can be disrupted at different points and to different degrees depending on the location and severity of the brain injury. Arousal reflects a general awareness of sensory stimuli and preparedness to respond (100). It is considered as a general state without a specific target or stimulus, analogous to cortical tone, which fluctuates during wakefulness or sleep. Deficits in arousal concern the energizing (quantitative) aspects of purposeful behavior rather than the directional (qualitative) aspects. Consequently, arousal deficits are characterized by lethargy and drowsiness. Clinically the presentation may be confused with loss of drive and these terms are sometimes (incorrectly) used interchangeably.

The term “drive” has a more ambiguous meaning but should be considered to refer to lack of purpose to act in people who appear fully alert. Wood and Eames (101) describe drive as a basic physiological process, a property of the organism which provides the impetus for behavior. Whilst intrinsic to the individual, drive is also stimulated by the environment, different external cues activating drive to a variable extent. Drive-based disorders often underlie descriptive terms, such as anergia or adynamism. Psychic akinesia refers to a loss of spontaneous mental processing in the context of normal externally-triggered mental function. It has been termed auto-activation disorder (102) and indicates a form of higher-order deficit in the generation of ideas, more fundamental and separate from motivation, and occurs in the context of normal intelligence (103). Brown and Marsden (104) described abulia as a kind of psychic akinesia, noting that this condition is characterized by apathy but not low mood. Abulia consists of a symptom cluster which includes aspontaneity and slowness and rigidity of movement. However, there is less consensus amongst practitioners on whether it should be understood as a primary disorder of motivation or a disconnection between the desire to act and the ability to act on that intention (105).

The construct of motivation is considered by many to be at the highest level of purposeful behavior, usually defined as an incentive or reason for acting. Whereas arousal is a general physiological state, with drive representing the physiological basis for goal-directed behavior, motivation is a more complex psychological construct that encompasses diverse cognitive and affective factors. Diminished motivation is fundamental to Marin's (106) influential concept of apathy, which he characterized as an impairment in goal-directed thoughts and behaviors, a loss of interest, combined with indifference to planning or setting goals, plus a lack of effort to achieve simple goals set by others.

Disorders that reflect diminished motivation are also linked to executive dysfunction. For instance, the pursuit of goals requires the capacity to identify, evaluate and prioritize goals, but also the ability to ignore external distractions, suppress other internal drives and initiate purposeful behavior. A deficit in any of these processes can result in similar psychosocial consequences but careful analysis will yield more information about underlying difficulties. To assist clinical assessment, Oddy et al. (107) proposed a five-stage model incorporating physiological, motivational and executive components as a basis for conceptualizing and treating a wide range of motivational disorders after TBI.

### Neural Basis of Drive and Motivation Disorders

Neurological disorders of arousal and drive are commonly associated with damage to brainstem and basal forebrain structures or cortico-subcortical networks involving the thalamus. The brainstem Ascending Reticular Activating System (ARAS) connects the thalamus, hypothalamus and basal forebrain, with the brainstem and forebrain providing important cholinergic inputs to thalamic nuclei. Central thalamic neurons are thought to be involved in supporting a distributed network which maintains neuronal activity through cortico-striatopallidal-thalamocortical circuits (108). These thalamic neurons are involved in responses to situational change, such as increased cognitive demand and stress. Damage to these circuits results in impairment of arousal regulation and forebrain activation underpinning goal-directed behavior.

The neural basis of motivation is less well-understood, with research largely focussing on apathy in the context of progressive neurological conditions. Such research indicates a complex relationship between psychological factors such as subjective value and outcome expectancies mediated by fronto-subcortical circuits linked to reward sensitivity and emotional state and ultimately dependant on effective motor networks. The role of mesolimbic dopamine for translating motivation into action has long been recognized and notions of dopamine depletion underlie many attempts to explain impairments of drive and motivation. Damage to the dopamine-rich ventromedial prefrontal cortex is linked to a range of deficits in sensitivity to reward, emotion-based learning and decision making. In addition, a wide range of subcortical structures including the anterior cingulate, hippocampus, insula, striatum and amygdala have been implicated in mediating stimulus-reward associations that drive purposeful behavior. Evidence for the role of specific brain structures in neurobehavioral disorders of diminished motivation and their psychosocial sequelae after traumatic brain injury has been reviewed recently by Worthington and Wood (12).

### Psychosocial Impact of Drive and Motivation Disorders

Apathy and diminished motivation have been associated with a wide range of negative consequences, such as poor recovery and rehabilitation outcome (109, 110), loss of social autonomy (111, 112), loss of vocational opportunities, with obvious financial implications (113), risk of cognitive decline (114), caregiver distress (115), poor quality family life (115), and poor social reintegration (112).

#### Psychological Impact

In understanding the impact of diminished motivation on the individual with brain injury it is important to consider how this is defined and measured in order to take account of confounding variables such as depression. Most research concerns apathy. Estimates vary from 20 to 70 per cent (113, 116). For example, (117) found that apathy (mixed with depression) occurred in 60 per cent of their sample, whilst Andersson et al. (118) found that almost half of their TBI sample had significant degrees of apathy. Apathy often occurs alongside other problems such as depression, fatigue and dysthymia, leading to difficulty establishing whether apathy is a primary disorder reflecting neurological damage or part of a broader set of symptoms of an underlying psychological disorder. Marin (119) argued that apathy or diminished motivation as a disorder was not caused by emotional distress and it would be illogical to refer to someone as *suffering* from apathy. Levy et al. (120) argued that apathy or lack of motivation should not be assumed to reflect depression, with the latter being characterized by sadness, hopelessness and worthlessness. Similarly, Marin and Wilkosz (121) highlighted that depression, but not apathy, is characterized by dysphoria.

Psychologically, the impact of apathy on the individual is often very limited, reflecting the impact of emotional blunting with little impetus for change. People with apathy often express indifference to their situation; they may know what should be done, and they are aware of their failings, but do not exhibit the frustration or distress that usually accompanies such insights. Instead, it is their loved ones who express these reactions when faced with an apathetic relative.

#### Impact on Daily Living

Although not typically associated with personal psychological distress, disorders of drive and motivation can severely undermine the ability to care for oneself and function autonomously, increasing dependence, even in people with preserved intellect. This is often mistaken for wilful behavior, such as laziness or obstinacy, or a form of self-neglect related to depression, when it is really a lack of impetus for behavior. This was neatly summarized by Pachalska et al. (122) when distinguishing between the inability to complete a task due to poor decision making and errors, from those who “*fail in task performance because they never actually begin: rather than make wrong decisions they make no decision at all, even though in most cases these patients can describe in detail what needs to be done*” (p. 2)

Shallice et al.'s (123) description of their case DN is typical: “*He is untidy. Shaving, changing his clothes or undergarments, washing his hair and having his hair cut are only carried out when his wife tells him. He hardly ever spontaneously tackles any domestic chores … if his wife is out, he normally leaves the preparation of a meal to his 10 year-old son*” (pg. 730).

However, even when a person may lack the facility for acting spontaneously, they could still be stimulated into action by situational triggers. Someone may lack spontaneity and sit about aimlessly whenever left alone but will readily engage in activity if prompted, for example by a text message or telephone call. Sometimes the action is only triggered in the presence of another person to cajole or model the behavior, and is only maintained by the same level of assistance.

People who lack internal motivations may be especially susceptible to cues in their environment. Lhermitte et al. (124) described an environmental dependency syndrome in which he postulated that the person's decision to act was not one they made for themselves. He described several such cases, including a lady who was apathetic all day but who could prepare a meal perfectly once in the kitchen if asked to by her husband, “*mental inertia and apathy played a part in the sense that the patients were powerless in the face of influences from the outside world*” (pg. 342).

Luria et al. (125) similarly described a ‘pathological inertia’ linked to frontal lobe lesions, “*Clinicians are well aware of the fact that patients of this group cannot look after themselves; even if hungry they will not ask for food and will not, of their own accord, reach for it. Bread must be put into their hand or they must be given a spoon in order to trigger the act of eating*” (p. 237). Consequently, the support needs (and care burden) of people with diminished drive and motivation are often considerable in order to fulfil their potential for adaptive living which they are incapable of doing when left to their own devices.

#### Impact on Relationships

Most research on the impact of TBI on relationships does not address the effects of diminished drive and motivation in isolation but it is frequently cited as a key factor. In one early study, aspontaneity after brain injury was one of the most common complaints of relatives (126). Lack of spontaneity was also evident in the author's 10-15 year follow up in 18 of 35 cases (127). McKinlay et al. (7) reported that the most frequent complaints of relatives at three, six and 12 months post-injury were slowness, tiredness and irritability, all of which were reported in at least two-thirds of respondents at each stage of recovery. Rosenbaum and Najenson (45) reported that partners felt depressed and isolated, with depressed mood amongst wives correlated with reduction in the brain-injured partner sharing childcare and their own child-like dependency. Subsequently, in a series of structured interviews inertia was reported by 89 of 98 “collateral” informants (partners, caregivers, colleagues) as a significant problem (128). This suggests that lack of drive and motivation is a major factor disturbing equilibrium in a relationship.

Apathy is more commonly reported by relatives (129) and clinicians (118) than brain injured persons themselves (130) but is often misinterpreted. Efforts made to energize individuals who are apathetic can elicit an aggressive reaction (131). This adds significantly to the stress of living with a family member who exhibits diminished motivation, whilst their failure to partake in marital or family activities can leave the whole family feeling estranged. This was explored recently using the Apathy Inventory. Arnould et al. (129) measured apathy, care burden and psychosocial functioning in close relatives of 68 adults with severe TBI (30 parents, 27 spouses and 5 siblings). Results showed that aspects of apathy (emotional blunting, lack of interest, lack of initiative) were linked to relatives' subjective care burden whereas poor psychosocial functioning was associated specifically with emotional blunting.

#### Impact on Employment

Disorders of drive and motivation have not been systematically studied as barriers to employment in their own right but clinicians are familiar with the problems they cause for vocational reintegration. In their early study of social recovery of 54 severe closed head injured adults, Oddy and Humphrey (132) reported that all but six had returned to work within 2 years, with physical disability considered a greater impediment than personality changes. This is not surprising if one considers that physical deficits are usually more apparent and can have more obvious constraints on ability to work. Getting back to work is not the same as being able to maintain employment however and it is likely that ultimately, job-limiting changes in temperament take time to emerge. These authors also point out that although ostensibly people were back in the same job employers reported subtle alterations in their expectations. This is a key point: the work environment may be sufficiently structured to allow people with some residual motivation to function adequately, whilst supportive employers may be able to adjust the work role sufficiently to accommodate difficulties. For example, an employer may downgrade their responsibility, provide additional manual or clerical support, or incentivize goal achievement.

Conversely, the workplace can be an unforgiving environment in which vulnerabilities like reduced drive, initiative and spontaneity are exposed in public. Unsympathetic colleagues may resent someone not pulling their weight, and financial losses can follow. For these reasons, disorders of drive and motivation often underlie workplace difficulties or failure to make a successful return to employment, although they can easily be mistaken, especially during the course of litigation where there may be a disincentive to return to work. These difficulties can add to the emotional and financial burden of a family coping with the aftermath of brain injury.

## Developmental Implications

It is self-evident that if NBD's, of the kind elucidated above, occur during childhood, before maturational development is complete, then the process of development will be undermined and there is likely to be in insidious impact on the individual once middle teenage years are reached.

Neurons within the ventromedial prefrontal cortex (vmPFC) encode the emotional value of sensory stimuli in a way that is not only necessary for the normal generation of emotions, but how emotions develop and guide appropriate social behavior (133–136). Therefore, the developmental timing of TBI and the concurrent onset of deficits of emotional experience and expression including the lack of empathy described above (see section—“Failure of social cognition”), may also lead to more profound and serious psychosocial difficulties.

### Impact on Social Judgement

Shamay-Tsoory et al. (137) and Shamay-Tsoory and Aharon-Peretz (138) proposed that the right vmPFC was necessary for the development of empathy which, in turn, is intrinsic to our understanding the emotions of others (social judgement). These studies noted that patients with lesions in the right vmPFC had a selective impairment that suggested a double dissociation between cognitive and affective theory of mind. Koenigs et al. (139) found that six patients with focal bilateral damage to the vmPFC exhibited an abnormally “utilitarian” pattern of judgements on moral dilemmas and reduced social emotions (such as compassion, shame and guilt) which are closely associated with the development of moral values (140, 141). An absence of emotional awareness has been associated with poorly regulated anger and low frustration tolerance without any loss of general intelligence, logical reasoning, or declarative knowledge of social and moral norms (142–144). Koenigs et al. (139) concluded that vmPFC damage diminishes the typical aversive affective response to harmful actions, reducing the impact of emotional control in the development of normal judgements to distinguish right from wrong. The affective functions of the vmPFC therefore seem necessary for normal moral judgement (145), bringing affect to bear on decision-making processes (146), reinforcing the key role of affect in the development of moral judgement (147).

### Impact on Social Learning and Moral Development

Koenigs et al. (139) hypothesized that the vmPFC plays a critical role in the development of emotional learning and social or moral knowledge during childhood. Taber-Thomas et al. (148) also argue that the risk of moral impairment depends not just upon the location of brain injury but the stage of maturational development when the injury occurs. Many social skills emerge early in life (149), and represent important milestones in moral development (150, 151). The maturation of moral judgement, transitioning from selfish to social, has long been theorized as an essential marker of typical social and moral development (152, 153). Mosch et al. (154) and Trauner et al. (155) both found that damage acquired earlier in development leads to less severe cognitive outcomes but more severe impairments in social and moral reasoning (140, 148). Consequently, dysfunction of the vmPFC (on the neural side) and impaired empathy (on the psychological side) may play central roles in psychopathy, a neurodevelopmental disorder hallmarked by callous, manipulative, egocentric and impulsive antisocial behavior (156, 157).

Anderson et al. (140) reported on two cases of individuals with vmPFC injury acquired during early childhood who exhibited a lack of empathy and amoral behavior. The two patients were injured before 10 years of age and were 20 and 23 years old at time of follow up. Whilst their intellect, memory and language had developed normally, they exhibited impaired decision making and were unable to make realistic plans for the future. Their behavior was characterized by physical and verbal abuse, sexual irresponsibility, a lack of empathy for others and an egocentric perspective on the world. They failed to acquire social and moral knowledge and, as adults, exhibited moral reasoning appropriate for a 10 year old with no sense of guilt, remorse, or moral responsibility. They exhibited—“*Behaviour akin to that of a psychopath*” (p. 1035), with asocial behavioral patterns more severe than those typically observed in patients with adult-onset vmPFC lesions.

The vmPFC may therefore be critical for the acquisition and maturation of moral faculties. It has been argued that a lack of moral development could be attributable to impaired aversive learning to self-serving moral transgressions based on complex social-emotional reinforcement contingencies [e.g., punishment for selfish behavior that is hurtful to others; (158–160)]; leading to an immature, abnormally egocentric moral sensibility. Damage that occurs later in life may not affect this early phase of moral development so severely, even though vmPFC damage acquired at any point in life is likely to impact upon the ability to integrate social-emotions (e.g., an aversion to harming others or to performing selfish actions) into reasoning about novel, complex moral situations (139, 148, 161, 162).

### Failure of Moral Development and Risk of Offender Behavior

The evidence relating to abnormal moral development and sociopathic patterns of behavior after injury to the vmPFC in childhood may explain why such a high number of offenders in custody have a history of head/brain injury. Williams (163) reported prevalence rates for TBI in young incarcerated male offenders (average age 16 years) as high as 60%, while McMillan et al. (164) recently found that the prevalence of hospitalized head injury in prisoners (24.7 per cent; 1080/4,374) was significantly higher than a matched general population sample (18.2 per cent; 2394/13122). In a systematic review of youth offending, Hughes et al. (165) reported prevalence rates of brain injury amongst incarcerated youth of between 16.5 and 72.1 per cent. In addition, Pitman et al. (166) showed that almost half (47 per cent) of their sample of 613 adult male prisoners reported a history of TBI when screened on admission to prison. The majority (70 per cent) of offenders reported receiving their first injury prior to their first offense.

These studies reinforce the notion that TBI may be a risk factor for offending, based on an assumption that injury acquired during an early stage of development results in a subsequent failure of emotional awareness, leading to a lack of empathy and a failure of moral judgement, the consequences of which are diminished social cognition, poor social-interpersonal communication skills, poor control over the need for gratification, and an absence of guilt or responsibility about how their “needs” are gratified. Consistent with this, numerous studies have shown that adults who sustained TBI in childhood are significantly poorer at emotion perception than healthy controls, exhibit a greater frequency of externalizing behaviors, have poor pragmatic communication ability, experience greater behavioral problems (i.e., emotional lability, aggression, disinhibition), and get into more trouble with law enforcement (167).

## Conclusion

To conclude, the way NBD presents clinically and socially across individuals can vary considerably because of the range of injury-related, personal and social factors, as well as the varying contributions made by cognitive, behavior and personality changes. Demographic factors, employment status, social and cultural factors, plus pre-injury psychiatric history and individual coping styles illustrate the complex interactions between factors that determine psychosocial outcome after brain injury, and how these need to be incorporated into a comprehensive programme of rehabilitation to support recovery. However, what is clear is that the presence of NBD after brain injury undermines social independence and can prevent survivors from achieving their full recovery potential. It has an adverse impact on a broad range of psychosocial functions and acts as a burden to both families and caregivers, potentially leading to increased social isolation, and, in more serious cases, gravitation to institutional placements for management purposes. In addition, as many social skills emerge early in life and represent important milestones in moral development, the age at which injury occurs should not be underestimated as an important consideration, as it can lead to further negative impacts, including an increased risk of offending. Therefore, more work on the often subtle but insidious nature of NBD during early maturational development is needed.

## Author Contributions

Each author was responsible for producing a first draft of a key section. CW social cognition. NA aggressive behavior. AW disorders of drive/motivation. RW developmental timing. CW and RW shared editorial responsibility throughout the drafting process. Final amendments were made by CW. All authors gave approval for submission, contributed significantly to the article, and are responsible for its contents.

### Conflict of Interest

The authors declare that the research was conducted in the absence of any commercial or financial relationships that could be construed as a potential conflict of interest.
